# Survey and Rapid Detection of *Bordetella pertussis* in Clinical Samples Targeting the BP485 in China

**DOI:** 10.3389/fpubh.2015.00039

**Published:** 2015-03-05

**Authors:** Wei Liu, Yinghua Xu, Derong Dong, Huan Li, Xiangna Zhao, Lili Li, Ying Zhang, Xiao Wei, Xuesong Wang, Simo Huang, Ming Zeng, Liuyu Huang, Shumin Zhang, Jing Yuan

**Affiliations:** ^1^Institute of Disease Control and Prevention, Academy of Military Medical Sciences, Beijing, China; ^2^National Institutes for Food and Drug Control, Beijing, China

**Keywords:** BP485, *B. pertussis*, LAMP, sensitivity, specificity, rapid diagnosis, prevalence

## Abstract

*Bordetella pertussis* is an important human respiratory pathogen. Here, we describe a loop-mediated isothermal amplification (LAMP) method for the rapid detection of *B. pertussis* in clinical samples based on a visual test. The LAMP assay detected the BP485 target sequence within 60 min with a detection limit of 1.3 pg/μl, a 10-fold increase in sensitivity compared with conventional PCR. All 31 non-pertussis respiratory pathogens tested were negative for LAMP detection, indicating the high specificity of the primers for *B. pertussis*. To evaluate the application of the LAMP assay to clinical diagnosis, of 105 sputum and nasopharyngeal samples collected from the patients with suspected respiratory infections in China, a total of 12 *B. pertussis* isolates were identified from 33 positive samples detected by LAMP-based surveillance targeting BP485. Strikingly, a 4.5 months old baby and her mother were found to be infected with *B. pertussis* at the same time. All isolates belonged to different *B. pertussis* multilocus sequence typing groups with different alleles of the virulence-related genes including four alleles of *ptxA*, six of *prn*, four of *tcfA*, two of *fim2*, and three of *fim3*. The diversity of *B. pertussis* carrying toxin genes in clinical strains indicates a rapid and continuing evolution of *B. pertussis*. This combined with its high prevalence will make it difficult to control. In conclusion, we have developed a visual detection LAMP assay, which could be a useful tool for rapid *B. pertussis* detection, especially in situations where resources are poor and in point-of-care tests.

## Introduction

*Bordetella pertussis*, a Gram-negative bacterium first reported in 1906 (1), is an important human-specific respiratory pathogen that causes whooping cough (pertussis) in humans. Although the morbidity and mortality of *B. pertussis* have been dramatically reduced since the introduction of Pw and Pa vaccines into the routine immunization schedule of infants, pertussis is considered as the most prevalent vaccine-preventable disease (2). It was revealed that neonates are now commonly infected by adolescents and adults, before transmitting whooping cough further to infants and other groups. This change in transmission pattern from “child to child” to “adult to child” may be the major cause for the rising incidence of pertussis, especially in developed countries with high vaccination coverage (3–7). In China, pertussis is a reportable infectious disease, and although the number of reported cases has been decreasing, pertussis remains endemic. A diagnosis is made on the basis of clinical symptoms, not using diagnostic tests such as culture, PCR, and serologic analysis. Therefore, the reported low incidence may, in fact, be due to substantial underreporting caused by misdiagnosis. However, timely and accurate diagnosis is necessary for controlling the disease.

Various diagnostic tests have been established, such as culture (8), PCR (9, 10), real-time PCR (11, 12), and enzyme-linked immunosorbent assay (13). Methods based on molecular biology or immunology have become universal and are increasingly replacing culture-based methods, as they are more rapid and sensitive. However, all of the above methods are time-consuming and require specialized equipment.

Loop-mediated isothermal amplification (LAMP) is a simple and cost-effective nucleic acid amplification method, which can be applied to rapidly detect *B. pertussis*. A LAMP assay can be completed within 1 h at a constant temperature using simple instruments. Further advantages include its high sensitivity and specificity. LAMP has previously been used to detect *B. pertussis* (14–17). Previously described PCR and LAMP assays have typically used IS*481*, which is present in more than 200 copies in the *B. pertussis* genome, as the target sequence (18, 19). However, because *B. holmesii* and some *B. bronchiseptica* strains contain the same IS*481* elements, these assays lacked specificity (20, 21). In 2008, Probert et al. compared the genomes of *B. pertussis*, *B. bronchiseptica*, and *B. parapertussis*, and tested the BP283 and BP485 sequences for the specific detection of *B. pertussis* using real-time PCR. They reported an improvement in specificity over assays targeting only IS*481* (22). Our study is the first to use BP485 as the target sequence in a LAMP assay. The purpose of the present study was to develop a rapid and simple test for *B. pertussis* detection, based on LAMP technology, which requires only basic equipment and allows immediate interpretation of results by visual inspection.

## Materials and Methods

### Bacterial isolates, identification, and MLST typing

Thirty-four bacterial strains were used for specificity analysis, which are listed in Table 1. Three *B. pertussis* vaccine strains were tested, including strain 18530, which originated from United States, strain P3s10, which has been used to produce Pw vaccines since the early 1960s, and strain CS, which has been used to produce Pa vaccines since 1995. Paired sputum and nasopharyngeal swabs were collected from 105 hospitalized patients using rayon-tipped swabs (Copan Diagnostics; Corona, CA, USA) between the spring of 2010 and spring of 2014. The patients (age range, 14 days to 35 years; median, 1.33 years; 52 females, 50 males) came from rural areas of China and had been treated for coughs and pneumonia for more than 7 days. Ten pairs of sputum samples and nasopharyngeal swabs from healthy people were also collected as controls. One swab or sputum sample was immediately placed in Regan-Lowe transport medium for culture and the other was stored at −70°C in an empty sterile tube for bacterial nucleic acid isolation.

**Table 1:** **Bacterial strains used in this study**.

No.	Strain	Source
1	*B. pertussis* ATCC18530	Our microorganism center
2	*B. pertussis* ATCC58003	Our microorganism center
3	*B. pertussis* ATCC53894	Our microorganism center
4	*B. parapertussis* CMCC 58302	Our microorganism center
5	*B. parapertussis* ATCC 15237	Our microorganism center
6	*B. parapertussis* ATCC BAA-587	Our microorganism center
7	*B. parapertussis* ATCC 53893	Our microorganism center
8	*B. bronchiseptica* ATCC BAA-588	Our microorganism center
9	*B. bronchiseptica* CMCC 58401	Our microorganism center
10	*B. bronchiseptica* ATCC 4617	Our microorganism center
11	*B. holmesii* ATCC 51541	Our microorganism center
12	*B. avium* ATCC 35086	Our microorganism center
13	*B. hinzii* ATCC 51730	Our microorganism center
14	*B. petrii* ATCC BAA-461	Our microorganism center
15	*Corynebacterium diphtheriae* CMCC 38001	Our microorganism center
16	*Haemophilus influenzae* CMCC 58534	Our microorganism center
17	*Betahaemolytic streptococcus* group A CMCC 32213	Our microorganism center
18	*Streptococcus pneumonia* CMCC 31001	Our microorganism center
19	*Neisseria meningitides* group B CMCC 29022	Our microorganism center
20	*Neisseria meningitides* group C CMCC 29026	Our microorganism center
21	*Neisseria meningitides* group Y CMCC 29028	Our microorganism center
22	*Neisseria meningitides* group A CMCC 29202	Our microorganism center
23	*Mycobacterium tuberculosis* 4368	Our microorganism center
24	*Neisseria meningitides* NM29019	Our microorganism center
25	*Streptococcus pneumonia* SP112-07	Our microorganism center
26	*Legionella pneumophila* LP9135	Our microorganism center
27	*Haemophilus influenzae* M5126	Our microorganism center
28	*Klebsiella pneumonia* 46117	Our microorganism center
29	*Vibrio parahaemolyticus* 5474	Our microorganism center
30	*Salmonella enteritidis* 50326-1	Our microorganism center
31	*Salmonella paratyphi A* 86423	Our microorganism center
32	*Shigella flexneri* 4536	Our microorganism center
33	*Shigella sonnei* 2531	Our microorganism center
34	*EIEC* 44825	Our microorganism center

*Bordetella pertussis* strains were cultured on Bordet–Gengou agar supplemented with 15% defibrinated sheep blood for 4–5 days at 37°C. Species identification was carried out using the Phoenix Automated Microbiology System (BD Diagnostic Systems) and matrix-assisted laser desorption ionization time-of-flight (MALDI-TOF) mass spectrometry. Genomic DNA was extracted from all samples using TIANamp Bacteria DNA Kit (TIANGEN Co., Ltd., Beijing, China). The sequences of 16S ribosomal DNA (rDNA) and BP485 were validated by PCR-based sequencing and showed 100% identity with those previously reported.

Seven housekeeper genes including *adk*, *fumC*, *glyA*, *tyrB*, *icd*, *pepA*, and *pgm* were detected by PCR. The allele number for each gene was assigned on the basis of the information in the *B. pertussis* MLST database^1^. Moreover, the strains were screened for the presence of 5 virulence-related genes (*prn*, *ptxA*, *tcfA*, *fim2*, and *fim3*) by PCR as previously reported (23).

### Primer design for LAMP

The sequence of BP485 (Accession number: CP002695.1) was downloaded from NCBI GenBank database and further analyzed by Primer Explorer Version 4^2^. Five primer sets were designed and synthesized commercially (Sangon Biotech Co., Ltd., Shanghai, China).

### LAMP assay

Loop-mediated isothermal amplification assays were carried out using the Loopamp DNA Amplification kit (Eiken Chemical Co., Ltd., Tochigi, Japan). Briefly, the LAMP amplification system in a final volume of 25 μl contained 12.5 μl reaction mixture, 1 μl *Bst* DNA polymerase, 2 μl template for real-time turbidimeter, and another 1 μl calcein/Mn^2+^ solution for visual detection. Primers were used at a concentration of 40 pmol for FIP and BIP, 20 pmol for LB and LF, and 5 pmol for F3 and B3. The reaction mix was overlaid with a sealing agent mainly comprised wax to prevent cross contamination of samples by aerosol. During the amplification, the protectant melted and became liquid without disturbing the reaction, avoiding volatilization of amplified products after reaction, and the protectant solidified as the temperature in the tubes decreased (Patent: ZL201210371448.5 in China).

Amplification was monitored using two methods, real-time changes in turbidity or visual detection of a color change. Pyrophosphate ions are released during LAMP amplification that with the addition of magnesium ions (Mg^2+^) in the reaction buffer form a white magnesium pyrophosphate precipitate (24), which can be monitored every 6 s at 650 nm using a real-time turbidimeter, and the data are transformed into turbidity curves. For visual detection, 1 μl of calcein/Mn^2+^ fluorescent detection reagent was added to the reaction. LAMP amplification results in a green fluorescent emission as a result of magnesium ions forming a complex with calcein. The color change from orange to green when samples are positive is visible to the naked eye (25). Each experiment was performed at least three times.

### PCR assay

PCRs were setup using 12.5 μl of PCR MasterMix reagents (Tiangen Biotech Co., Ltd., Beijing, China), 1 μM BP-113F3 and BP-113B3 primers, and 2 μl of DNA template in a final volume of 25 μl. PCR was performed using the following cycling conditions: initial PCR activation, 95°C for 5 min; amplification, 40 cycles of 95°C for 30 s, 55°C for 30 s, and 72°C for 30 s; final extension, 72°C for 7 min. PCR products were visualized on a 1% agarose gel stained with ethidium bromide. Images were documented with a Gel Doc EQ imaging system (Bio-Rad).

## Results

### Optimization of LAMP assay

**Figure 1:**
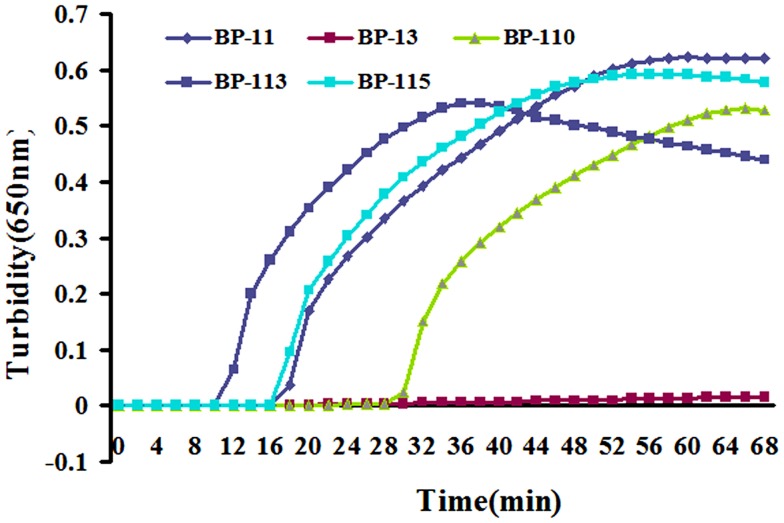
**Five designed primer sets were tested by real-time turbidimeter at 650 nm every 6 s**. BP-113 group possess optimal efficiency and was chosen to be final primers for LAMP assay.

BP485 was targeted for detection of *B. pertussis* by a LAMP assay for the first time. Five different primer sets were initially tested, four of which resulted in successful amplification as shown in Figure 1. The BP-113 primer set amplified the target sequence within the shortest time and was therefore chosen as the optimal primer set (Table 2).

**Table 2:** **Sequence of optimal primer set for LAMP assay**.

Primer	Length (bp)	Sequence (5′–3′)
BP113-F3	19	GCGATCTCGATGCTTGACG
BP113-B3	20	CCTCATCTTCGTTCAGCGAA
BP113-FIP	44	AGAAACAGTGGCTCGATGGCGTTTTTTCACTA TGGGCTGTCGTG
BP113-BIP	43	TTGATTGACAGGGCAATCCGGCTTTTGCGTGT TTTCCCCAGAG
BP113-LF	17	GTGCTTGACGTGACCGC
BP113-LB	19	AATAGCGCAGTCCGGCGTA

*bp, base pair*.

**Figure 2:**
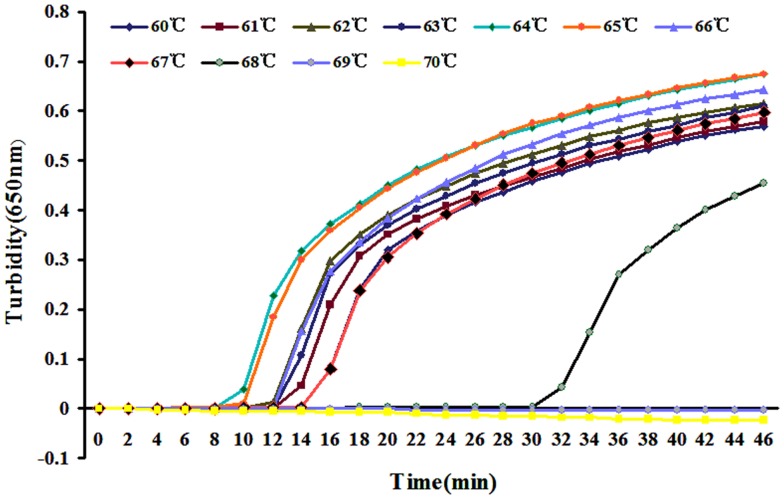
**Different temperatures for LAMP reaction were tested using real-time turbidimeter at 650 nm**.

Reaction temperatures from 60 to 67°C were compared for optimal amplification. Amplification efficiency was highest at 64°C (Figure 2) and was therefore used for later experiments.

### Specificity of LAMP assay

**Figure 3:**
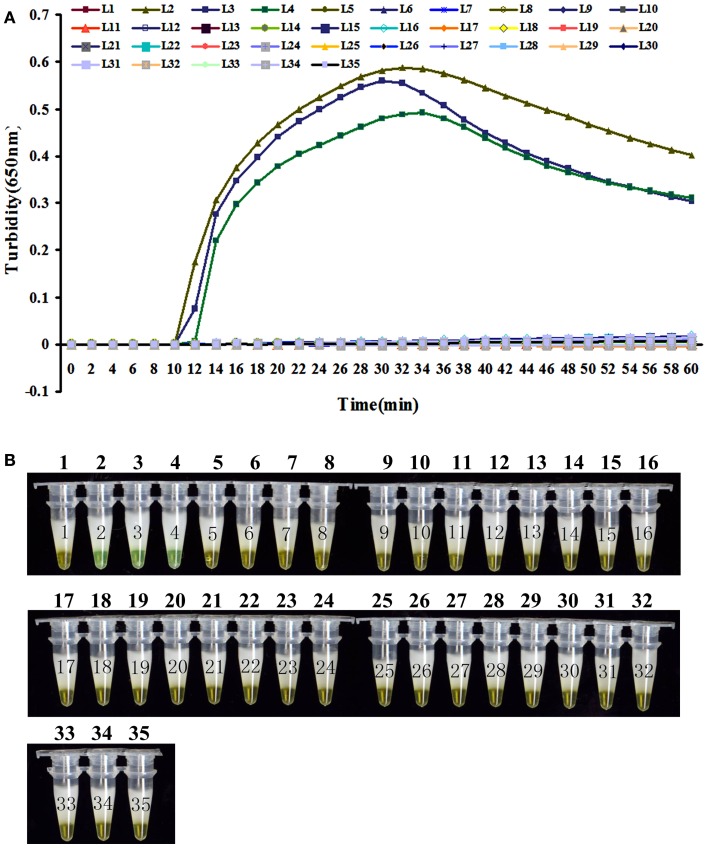
**The specificity detection of LAMP assay by real-time turbidimeter (A) or addition calcein to the reaction tube (B)**. Amplification was performed at 64°C for 60 min. 1, NC; 2, *B. pertussis* ATCC 18530; 3, *B. pertussis* CMCC 58003; 4, *B. pertussis* ATCC 53894; 5, *B. parapertussis* CMCC 58302; 6, *B. parapertussis* ATCC 15237; 7, *B. parapertussis* ATCC BAA-587; 8, *B. parapertussis* ATCC 53893; 9, *B. bronchiseptica* ATCC BAA-588; 10, *B. bronchiseptica* CMCC 58401; 11, *B. bronchiseptica* ATCC 4617; 12, *B. holmesii* ATCC 51541; 13, *B. avium* ATCC 35086; 14, *B. hinzii* ATCC 51730; 15, *B. petrii* ATCC BAA-461; 16, *Corynebacterium diphtheriae* CMCC 38001; 17, *Haemophilus influenzae* CMCC 58534; 18, *Betahaemolytic streptococcus group A* CMCC 32213; 19, *Streptococcus pneumonia* CMCC 31001; 20, *Neisseria meningitides group B* CMCC 29022; 21, *Neisseria meningitides group C* CMCC29026; 22, *Neisseria meningitides group Y* CMCC 29028; 23, *Neisseria meningitides group A* CMCC 29202; 24, *Mycobacterium tuberculosis* 4368; 25, *Neisseria meningitides* NM29019; 26, *Streptococcus pneumonia* SP112-07; 27, *Legionella pneumophila* LP9135; 28, *Haemophilus influenzae* M5216; 29, *Klebsiella pneumonia* 46117; 30, *Vibrio parahaemolyticus* 5474; 31, *Salmonella enteritidis* 50326-1; 32, *Salmonella paratyphi A* 86423; 33, *Shigella flexneri* 4536; 34, *Shigella sonnei* 2531; 35, EIEC 44825.

To evaluate the specificity of LAMP detection for *B. pertussis*, genomic DNA was extracted from *B. pertussis* ATCC18530, *B. pertussis* ATCC58003, *B. pertussis* ATCC53894, and 31 other pathogenic respiratory bacteria and tested using real-time turbidity or visual detection of color change as readouts. Both methods of analysis positively identified the *B. pertussis* isolates. All other strains, including the blank control, tested negative, indicating that the LAMP assay was specific to *B. pertussis* (Figure 3).

### Sensitivity of the LAMP assay versus PCR for *B. pertussis*

**Figure 4:**
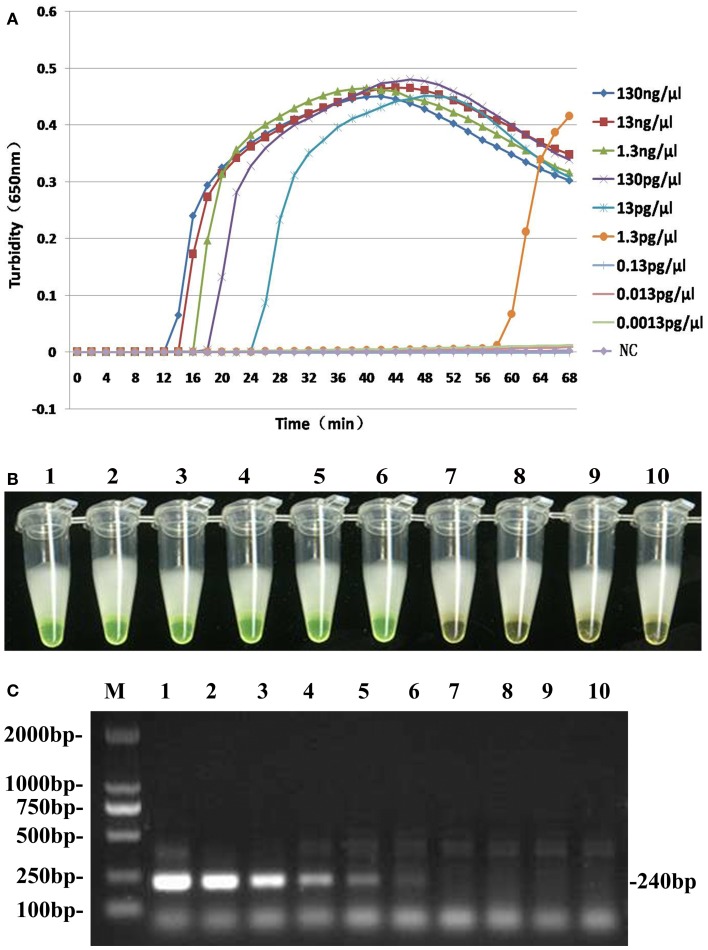
**Comparative sensitivities of both LAMP assay (A,B) and traditional PCR (C)**. Lane M: DL2000 Marker; Tubes 1–9 and lanes 1–9: 10-fold serial dilution pure genomic DNA extracted from *B. pertussis* (130 ng/μl to 0.0013 pg/μl); Tube 10 and lane 10: ddH_2_O as negative control.

To compare the detection limit of LAMP using either real-time turbidity measurements or color change with traditional PCR, 10-fold serial dilutions of genomic DNA extracted from *B. pertussis* ATCC18530 (130 ng/μl to 1.3 fg/μl) were tested. The results were shown in Figure 4. The detection limits of real-time turbidity and visual detection were both 1.3 pg/μl, which was 10-fold more sensitive than traditional PCR assay.

### Dissemination of *B. pertussis* in clinical

A total of 105 clinical sputum samples and nasopharyngeal swabs were collected for LAMP-based surveillance of BP485 from patients with suspected respiratory infections in China during 2010–2014. Ten pairs of sputum samples and nasopharyngeal swabs from healthy people were collected as controls. All clinical samples were analyzed by LAMP and PCR simultaneously. As shown in Table 3, of the 105 clinical samples, LAMP detected 33 positive samples while 23 were detected by PCR. Then, *B. pertussis* was successfully cultured from 12 samples, all of which had been positively identified by LAMP. The healthy control samples all tested negative in each of the assays.

**Table 3:** **Evaluation of LAMP and PCR for detection of *B. pertussis* in 105 clinical samples**.

Gene	Type of samples	No. of samples tested	No. of positive samples for assay		
			Culture	PCR	LAMP
BP485	Clinical nasopharyngeal swabs from patients	105	12	23	33
	Nasopharyngeal swabs from healthy people	10	0	0	0

The sequence analysis of the BP485 gene from the *B. pertussis* isolates confirmed conservation with the nucleotide sequences of reported genes. Multilocus sequence typing (MLST) analysis of *B. pertussis* showed that five of the seven housekeeping genes belonged to a single genotype, similar to reported global genotypes. However, the *adk* and *tyrB* housekeeping genes had two different genotypes belonging to type 1 and type 3, respectively.

To further characterize the virulence-related genes of the 12 isolated strains, the alleles of *ptxA*, *prn*, *tcfA*, *fim2*, and *fim3* were analyzed. All isolates had virulence-related genes belonging to different genotypes with all four *ptxA* alleles, six *prn* alleles, four *tcfA* alleles, two *fim2* alleles (*fim2-1* and *fim2-2*), and three *fim3* alleles (*fim3-1*, *fim3-2*, and *fim3-4*) present across the samples. Our data show that *B. pertussis* is widespread in respiratory infections, and the diverse genotypic features indicate on-going evolution.

## Discussion

Pertussis, also known as whooping cough, is an acute respiratory infectious disease caused by *B. pertussis*, and can cause severe disease, particularly in infants. Although vaccines are widely used in most countries, the morbidity of pertussis is rising, even in developed countries with high vaccination coverage. Remarkably, many adolescents and adults become infected despite having been vaccinated as infants as a result of waning antibody titers, which can result in further transmission to children. Unfortunately, conventional wisdom holds that pertussis is a pediatric disease, leading to substantial under-diagnosis in adolescents and adults. Pertussis is highly infectious, and therefore rapid and simple methods to aid diagnosis would be advantageous in controlling disease spread.

Several diagnostic techniques have been established for detecting *B. pertussis*, e.g., culture, serological assays, and, most commonly, PCR-based methods. These approaches are either time-consuming or costly and frequently require skilled technicians and precision instruments. They are therefore not suitable for situations where resources are limited and fast results are required such as for point-of-care assessments. Since its introduction in 2000, a nucleic amplification method known as LAMP (26) has been widely used in various fields, including clinical diagnosis, food safety, livestock breeding, and even determination of sex (27, 28). A number of reports indicated that LAMP had a better sensitivity and specificity for detecting pathogens than other commonly used methods. Here, we described a LAMP method for detecting BP485 of *B. pertussis* by color change.

Although many target genes, such as IS*481*, have been used to diagnose pertussis, we chose BP485, which, unlike IS*481*, is able to distinguish *B. pertussis* from *B. bronchiseptica* and *B. parapertussis* (22). Specificity evaluation was performed here using 3 *B. pertussis* strains and 31 homologous species by both real-time turbidimeter and visual color change methods. All *B. pertussis* strains tested positive and all 31 non-pertussis respiratory tract pathogens, including *B. bronchiseptica* and *B. parapertussis*, tested negative. These results demonstrated that LAMP targeting BP485 had a high specificity for the detection of *B. pertussis* and that using turbidity or color change as readouts gave comparable results. The sensitivity of LAMP to detect BP485 was compared with traditional PCR using 10-fold serial dilution of genomic DNA extracted from *B. pertussis*. The detection limit of the LAMP assay used here was 1.3 pg/μl, 10-fold higher than conventional PCR, in agreement with previous reports. Furthermore, LAMP can be completed in one hour using a simple thermostat, whereas a PCR run takes approximately 3 h and requires a relatively complicated thermal cycler. LAMP is therefore a more rapid and simple technique making it better suited to field use.

Two readouts were used for the LAMP assay to detect amplification products, real-time turbidity and visual observation of color change. Detection by color change was as sensitive and specific as real-time turbidity measurements. As this color change is visible to the naked eye, it would further simplify the assay. It is worth noting that the use of a wax sealant over the reaction mixture was essential to minimize cross contamination by aerosol, which frequently resulted in false positives.

Application of the assay to samples taken from hospital admissions of cough and pneumonia indicated that *B. pertussis* was prevalent in the community with over 30% of samples testing positive. The cases included a 35-year-old female patient from rural China who was admitted for treatment in the city hospital after suffering with cough and pneumonia for 14 days. Her baby had contracted whooping cough from her as a result of close contact. This was an example of increasing numbers of “adult to child” transmissions, which may be a major factor in the rising incidence of pertussis.

In conclusion, the visual LAMP assay developed here for detecting *B. pertussis* allows for positive samples to be identified by the naked eye immediately after amplification is completed. The visual method is as sensitive and specific as real-time turbidity measurements and is more sensitive than PCR. It is a rapid, simple, and low-cost method to diagnose *B. pertussis*, making it particularly useful for point-of-care testing where time and resources are limited.

## Author Contributions

JY and SZ helped conceive project and designed experiments. WL, YX, and HL performed and wrote the manuscript. XZ, LL, YZ, MZ, XW, XW, and SH designed and executed experiments. DD and LH helped to edit the manuscript.

## Conflict of Interest Statement

The authors declare that the research was conducted in the absence of any commercial or financial relationships that could be construed as a potential conflict of interest.

## Footnotes

^1^http://pubmlst.org/bordetella

^2^http://primerexplorer.jp/elamp4.0.0/index.html
